# Effect of a Mobile Health Intervention in the Management of Hypertension: Open-Label Cluster-Randomized Trial

**DOI:** 10.2196/72416

**Published:** 2025-12-24

**Authors:** Xiaoli Zhao, Qiang Zhao, Qinxian Yang, Yan Li, Xiaona Xing, Xiaoping Li

**Affiliations:** 1 Department of Cardiology, Sichuan Provincial People's Hospital, University of Electronic Science and Technology of China, No. 32, Section 2, Yihuan Road, Qingyang District, Chengdu, Sichuan, 610072, China, 86 18011159771; 2 Department of Ultrasound Medicine, Guangyuan Traditional Chinese Medicine Hospital, Guangyuan, Sichuan, China; 3 Department of Endemic Diseases and Vector Control, Guangyuan Lizhou District Center for Disease Control and Prevention, Guangyuan, Sichuan, China; 4 Department of Geriatrics, Guangyuan Wan Yuan Hospital, Guangyuan, Sichuan, China; 5 Department of Geriatrics, Chengdu Wenjiang District People's Hospital, Chengdu, Sichuan, China

**Keywords:** blood pressure monitoring, China, digital intervention, health education, hypertension control, hypertension management, online consultation, rural areas, smartphone, WeChat mini-program, mobile phone

## Abstract

**Background:**

Digital therapeutics represents a promising approach to support the management of hypertension. Rural regions in China face substantial challenges in hypertension prevention and management. Given the rapid growth in use of the internet and mobile technologies, particularly smartphones, we developed a user-friendly WeChat mini-program “E-controlled pressure (eKongya)” to assist village physicians in managing patients with hypertension.

**Objective:**

This trial aimed to investigate the efficacy of digital interventions for blood pressure (BP) control in patients with hypertension.

**Methods:**

This open-label cluster-randomized controlled study was conducted in 8 villages in China. Individuals with systolic BP (SBP) ≥140 mm Hg or diastolic BP (DBP) ≥90 mm Hg were recruited. Eight villages were randomly assigned in a 1:1 ratio to the digital intervention group or control group. The primary end point was the hypertension control rate at 24 weeks among the study participants. The secondary end points were the changes in mean SBP and DBP from baseline to 24 weeks. All analyses were performed using the full analysis set.

**Results:**

Between June and July 2024, a total of 95 participants were enrolled and allocated to the digital intervention group (n=48, 51%) or the control group (n=47, 49%). After 24 weeks, data were available from 87 (92%) participants, and the mean age was 63.8 (SD 9.7) years, with 48% (n=42) being female participants. The digital intervention group (25/44, 57%) had a higher percentage of participants with controlled BP compared to the control group (21/43, 49%), although this difference was not statistically significant (*P*=.60). Logistic regression analysis showed that the digital interventions did not significantly increase the hypertension control rate (odds ratio 0.73, 95% CI 0.31‐1.69; *P*=.46). In the intervention group, SBP decreased from 158.0 (SD 18.4) mm Hg at baseline to 137.5 (SD 13.0) mm Hg at 24 weeks and DBP decreased from 93.8 (SD 10.3) mm Hg to 85.3 (SD 11.6) mm Hg. In the control group during that same period, SBP decreased from 161.1 (SD 18.2) mm Hg to 139.6 (SD 13.2) mm Hg, and DBP decreased from 99.2 (SD 9.2) mm Hg to 83.4 (SD 12.1) mm Hg. After adjusting for baseline SBP or DBP, the mean change from baseline to 24 weeks was comparable between the digital intervention and control groups for both SBP (between-group difference −1.6, 95% CI −7.2 to 3.9; *P*=.56) and DBP (between-group difference 3.3, 95% CI −1.8 to 8.5; *P*=.21). No major program-related safety events occurred up to 24 weeks.

**Conclusions:**

Our study demonstrated that the digital interventions increased the hypertension control rate in rural areas, although this improvement was not statistically significant. Nevertheless, providing convenient BP measurements and health education to these patients notably enhanced hypertension control rates.

## Introduction

Hypertension is a leading cause of cardiovascular disease mortality in China, driven by its high prevalence and associated vascular risks [1]. According to 2018 statistics, of the 274 million adults aged 18 to 69 years with hypertension in China, an estimated 240 million have inadequate control of their condition [2]. From 2005 to 2018, a population-based study of 31 provinces in China showed that 2.67 million cardiovascular disease deaths were attributed to high systolic blood pressure (SBP), including 1.12 million from ischemic heart disease, 630,000 from ischemic stroke, and 580,000 from hemorrhagic stroke [3]. More concerning, the standardized prevalence, awareness, treatment, and control rates of hypertension in rural areas are 24.4%, 37.6%, 33.5%, and 9.5%, respectively [3]. Hypertension often presents with nonspecific clinical manifestations, leaving many patients asymptomatic, which contributes to a low rate of medical consultation. Rural regions face significant challenges in hypertension prevention and management, primarily due to low medical resources and limited self-care awareness among residents [4,5].

In light of these challenges, digital health solutions, particularly mobile apps, have emerged as promising tools to bridge the health care gap in underserved areas. The use of digital medical technologies may significantly improve patients’ understanding and management of the disease. Health care professionals could use digital data collection and analysis to provide a more comprehensive and accurate assessment of patients’ health, thereby supporting personalized treatment and care [6-8]. An extensive body of literature has demonstrated the effectiveness of digital interventions in managing hypertension, with some studies highlighting the use of mobile apps for patient management [9,10]. However, there is still a lack of a scientifically evaluated, collaboratively developed, sustainable, and widely adopted management tool in China.

Given the rapid growth of the use of the internet and mobile technologies, particularly smartphones, we developed a user-friendly WeChat mini-program “E-controlled pressure (eKongya)” to assist village physicians in managing patients with hypertension. WeChat is a widely used social media platform in China [11]. The “eKongya” WeChat mini-program is easily accessible via direct search within WeChat. In addition, this mini-program includes features for promoting self-monitoring of blood pressure (BP), hypertension education, and free online consultations, thereby helping to overcome health care access challenges in remote areas with limited medical resources. On the basis of previous literature, achieving BP control within 6 months was associated with a reduced risk of adverse outcomes compared to later control [12]. We hypothesized that digital interventions using the WeChat mini-program “eKongya” could improve hypertension control in individuals with hypertension over a 24-week period. Accordingly, this randomized controlled trial was designed to evaluate the role of digital interventions in hypertension management.

## Methods

### Overview

This is an open-label, cluster-randomized, controlled study that compared digital interventions for hypertension management to a control group. The study was conducted at 8 rural health care sites in China. We selected trial sites based on the following three criteria: a high prevalence of poorly controlled hypertension, geographic remoteness and limited access to health care, and the village physician’s willingness to participate. Patients were recruited between June and July 2024, and the follow-up began in December 2024 and ended in January 2025.

### Population

The inclusion and exclusion criteria are presented in Textbox 1.

Textbox 1. **Inclusion and exclusion criteria.**

Inclusion criteria

- Age ≥18 y
- Individuals, regardless of prior hypertension diagnosis, with a mean baseline blood pressure (calculated from the second and third readings): systolic blood pressure ≥140 mm Hg or diastolic blood pressure ≥90 mm Hg
- For participation in the digital intervention, individuals should be willing to engage in self-monitoring and be able to use the WeChat mini-program or have family members who could assist with its use

Exclusion criteria

- Patients with New York Heart Association (NYHA) Class IV heart failure
- Individuals who had experienced an acute cardiovascular event and stroke within the previous 3 mo
- Patients with advanced-stage cancer
- Individuals planning pregnancy, currently pregnant, or breastfeeding
- Individuals unable to use the WeChat mini-program even after training

### Randomization

Researchers screened eligible participants by reviewing medical records from 8 rural health care sites involved in the trial. Randomization (1:1) was performed using SPSS software (version 22.0; IBM Corp.) to randomly assign 8 villages to the digital intervention group or the control group. The randomization process included (1) generating a unique random number for each participating village; (2) sorting these random numbers in ascending order; and (3) allocating the first 4 villages to the intervention group, whereas the subsequent 4 villages were assigned as the control group based on the sorted random number assignment.

### Procedure

The village physicians contacted the identified eligible individuals via phone calls or WeChat to invite them to the local health care sites to learn about the study. Following this, the eligible people received printed education booklets containing information on the definition, causes, and basic treatment guidelines for hypertension (including lifestyle changes and medications), which were uniformly explained to them by the researchers. After resting for at least 15 minutes, the researchers measured their BP using an electronic BP monitor (Omron, HEM-7121). Three BP readings were taken at intervals of 1 minute. Those who met the criteria and wished to participate were asked to provide informed consent (Multimedia Appendix 1), after which their baseline data were collected. Participants in the digital intervention group could directly enter their information via the WeChat mini-program. The collected baseline data included demographic characteristics, physical examination findings, medical and personal history, laboratory test results, medication usage, and psychological assessments (including the Generalized Anxiety Disorder 7-item scale (GAD-7) Anxiety Screening Scale, Patient Health Questionnaire-9 item (PHQ-9) Depression Screening Scale, and Pittsburgh Sleep Quality Index).

### Interventions

#### Intervention Group

**Figure 1.**
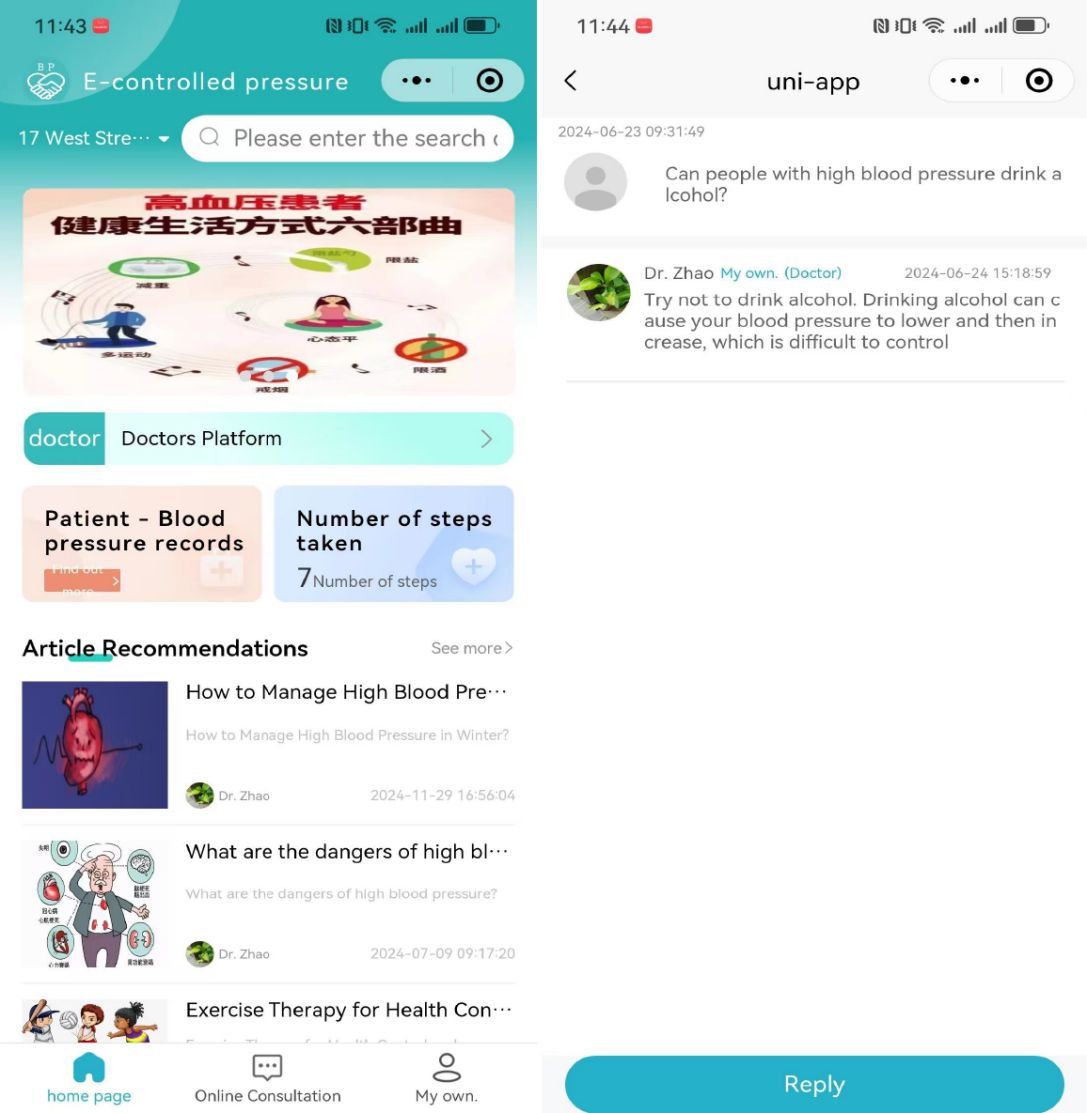
Screenshots of the (Chinese-language) smartphone WeChat mini-program. From left to right, the screenshots display the home page and an online consultation that enables patients to communicate with physicians.

Participants in the digital intervention group were provided with one-on-one guidance from the researchers on how to use the WeChat mini-program “eKongya” (Figure 1), which included entering baseline data, recording self-measured BP and lifestyle changes, accessing hypertension health education materials, and consulting a physician online (MultimediaAppendices 2  3). The hypertension health education materials provided information on the benefits of BP self-monitoring, health-related behaviors, and antihypertensive therapy, and addressed common concerns about medication side effects.

Participants received both face-to-face and online guidance on how to accurately measure BP using electronic monitors, ensuring they could perform self-measurements correctly. They were advised to measure their BP at least twice each morning for 7 days each month, with all data entered into the WeChat mini-program. If the average home BP remained within target levels for 3 consecutive months, the frequency of measurements could be reduced to twice on 3 days each month. If the average home BP exceeded the target for 2 consecutive weeks, investigators would inquire about potential causes and guide participants in adjusting their antihypertensive medication via the mini-program. If home BP readings were very high (>180/110 mm Hg) or very low (systolic <90 mm Hg), participants were advised to visit a local hospital.

According to the guidelines [13,14], antihypertensive treatment included guidance on healthy lifestyle practices and the initiation of pharmacotherapy. The choice and titration of medications for BP control followed these principles: unless there were specific clinical indications or contraindications, the following drug categories were used: angiotensin-converting enzyme inhibitors, angiotensin receptor blockers, angiotensin receptor-neprilysin inhibitors, calcium channel blockers (CCB), or diuretics. Preference was given to once-daily medications with a 24-hour effect. Treatment may involve monotherapy or combination therapy, with initial combination therapy preferably using angiotensin-converting enzyme inhibitors or angiotensin receptor blockers with CCB or thiazide diuretics. Fixed-dose combinations were also an option. If BP remained above the target, referral to a cardiovascular specialist was recommended for further evaluation. Medication dosages should be titrated every 1 to 2 weeks until reaching an appropriate dose.

#### Control Group

Participants received a printed education booklet, an electronic BP monitor, and face-to-face training on its use, without the need to engage with the WeChat mini-program. Upon enrollment, researchers offered tailored health guidance to each participant regarding BP control, including personalized recommendations for smoking cessation, a low-sodium diet, appropriate exercise, and comorbidity management. Importantly, these participants received conventional hypertension treatment. Furthermore, they were requested to voluntarily monitor and record their BP with the same frequency as the digital intervention group. At the end of the trial, data on self-monitoring collected from both patients and physicians were compiled.

In both the digital intervention and control groups, the specific medications administered were determined by licensed physicians.

### End Points

The targets of BP control were set according to the latest hypertension guidelines [13,14] (participants aged <80 years: <140/90 mm Hg; participants aged ≥80 years: <150/90 mm Hg; and those with diabetes, chronic kidney disease, coronary heart disease, or history of stroke, or transient ischemic attack: <130/80 mm Hg). The primary end point was the hypertension control rate at 24 weeks. The secondary end point was the change in mean SBP and DBP from baseline to 24 weeks.

### Follow-Up

This study aimed to investigate the effects of digital interventions on hypertension control. Two weeks after enrollment, participants were contacted by telephone to assess their ability to properly use the BP monitor. BP readings were not recorded at this time. Previous literature indicated that achieving BP control within 6 months was associated with a reduced risk of adverse outcomes compared to later control [12]. This time frame also allowed for sufficient adjustments in BP management. Therefore, follow-up visits were scheduled at 24 weeks post randomization and were conducted by researchers and village physicians. The main methods of follow-up included clinic visits or telephone calls, and participants’ BP was recorded.

### Sample Size

The sample size was calculated based on the following assumptions: based on previous studies, the hypertension control rate in the control group was set at 10% [14], whereas the estimated control rate for the digital intervention group was 40%. Referring to prior literature, assuming an intracluster correlation coefficient of 0.01 for the primary outcome [15,16], with a test power of 90% and a 5% type 1 error probability, a sample size of 42 patients per group was required to detect a difference in hypertension control rates between the digital intervention and control groups. Considering a potential 10% dropout rate, each group should initially include 47 participants (12 participants in each cluster).

### Statistical Analysis

All analyses were performed based on the full analysis set population. Patient characteristics at baseline were described using mean (SD) or median (quartiles) for continuous variables and number (proportion in percentage) for categorical variables. Normally distributed continuous variables were compared using Student *t* test with unequal variances, and continuous nonparametric variables were compared using Kruskal-Wallis test. Categorical variables were compared using the chi-square test. The primary end point, the hypertension control rate at 24 weeks, was compared between the digital intervention group and the control group using the chi-square test. To comprehensively assess the impact of digital interventions on the hypertension control rate, logistic regression analysis was conducted. The secondary end point was analyzed using analysis of covariance, adjusted for baseline BP, to avoid confounding the 24-week BP change. In addition, a 2-factor, 2-level repeated measures analysis was used to assess the impact of the digital interventions and the pre- and post-measurement time points on the change in mean SBP and DBP from baseline to 24 weeks.

Statistical analyses were performed using SPSS software (version 22.0; IBM Corp.) and R software (version 4.4.0; R Core Team), with the ggplot2 package (version 3.5.1; Posit, PBC) for creating figures. *P* values <.05 were considered statistically significant.

### Ethical Considerations

This study was reviewed and approved by the Medical Ethics Committee of the Sichuan Academy of Medical Sciences and Sichuan Provincial People’s Hospital, with the ethics review approval number: Ethics Review (Research) No. 47, 2024 (Multimedia Appendix 4). The trial was conducted in accordance with the Declaration of Helsinki and all relevant laws and guidelines in China. In addition, this study was reported in compliance with the CONSORT-EHEALTH (Consolidated Standards for Reporting Clinical Trials of Electronic and Mobile Health Applications and Online Telehealth) checklist (Checklist 1). All participants provided written informed consent before enrollment in the study. They retained the right to refuse participation or withdraw at any time during the research. No public reports of study results will disclose participants’ personal identities. As stipulated by the study protocol, and to compensate participants for their time and effort, each individual received an electronic BP monitor and a printed hypertension education booklet, provided free of charge. These items were intended for use throughout the study and to encourage ongoing health self-management thereafter. The combined approximate retail value of these provisions is US $35. This compensation plan, including the nature and value of the provided items, was explicitly reviewed and approved by the Medical Ethics Committee.

## Results

### Participant Characteristics

**Figure 2.**
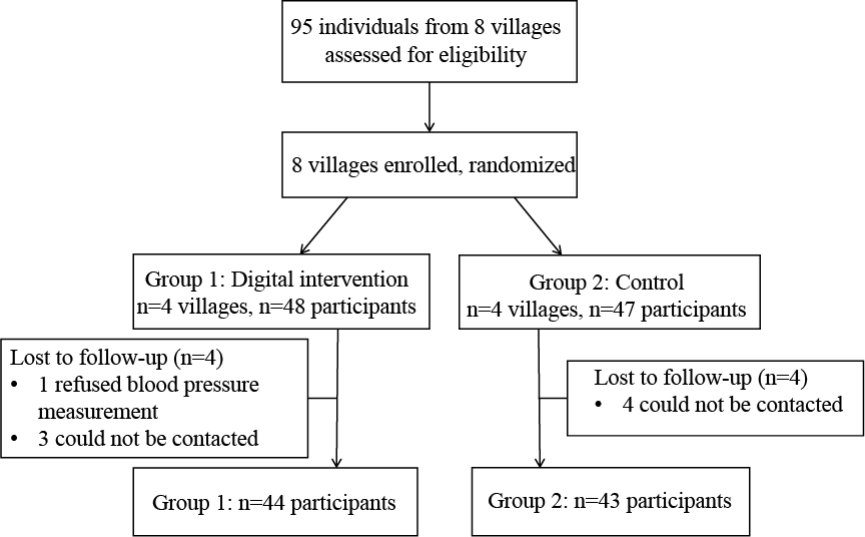
Study flow diagram.

Between June and July 2024, a total of 95 participants were enrolled and allocated to the digital intervention group (n=48, 51%) or the control group (n=47, 49%). Of these, 87 (92%) of 95 participants were followed for BP monitoring over 24 weeks (Figure 2). On average, study participants were aged 63.8 (SD 9.7) years, with 48% (n=42) being female participants, and the time since hypertension diagnosis averaged around 7.6 (SD 6.8) years. More than half of the participants had a primary school education and low incomes. In the digital intervention group, 91% (40/44) had a self-reported history of hypertension, and 77% (34/44) participants took antihypertensive medications, whereas in the control group, 81% (35/43) had a self-reported history of hypertension, and 77% (33/43) participants took medications. More than half of the participants were taking CCB (n=26, 59% in the digital intervention group and n=25, 58% in the control group); more details are presented in Table 1 and Multimedia Appendix 5.

**Table 1.** Baseline characteristics of study participants.

Characteristics of participants	Overall (N=87)	Control (n=43)	Intervention (n=44)	*P* value
Participants, n (%)	87 (100)	43 (100)	44 (100)	—^a^
Age (y), mean (SD)	63.8 (9.7)	66.0 (8.8)	61.8 (10.1)	.04
Gender (female), n (%)	42 (48)	17 (40)	25 (57)	.16
Race, n (%)				—
Chinese	87 (100)	43 (100)	44 (100)	
Other	0 (0)	0 (0)	0 (0)	
BMI (kg/m^2^), mean (SD)	23.3 (3.1)	22.7 (3.2)	23.7 (2.9)	.16
Systolic BP^b^ (mm Hg), mean (SD)	159.5 (18.2)	161.1 (18.2)	158.0 (18.4)	.43
Diastolic BP (mm Hg), mean (SD)	96.5 (10.0)	99.2 (9.0)	93.8 (10.3)	.01
Pulse (times per minute), mean (SD)	74.4 (11.2)	75.8 (10.6)	73.3 (11.6)	.33
Education, n (%)				.02
Primary school or lower	65 (75)	37 (86)	28 (64)	
Junior high school	15 (17)	6 (14)	9 (21)	
High school or higher	7 (8)	0 (0)	7 (16)	
Income, n (%)				.83
<¥1000 (US $141)	46 (53)	24 (56)	22 (50)	
¥1000‐¥3000 (US $141-$422)	29 (33)	13 (30)	16 (36)	
>¥3000 (US $422)	12 (14)	6 (14)	6 (14)	
Medical insurance coverage, n (%)				<.001
Residential health insurance	66 (76)	31 (72)	35 (80)	
Employees health insurance	9 (10)	1 (2)	8 (18)	
Other	12 (13)	11 (26)	1 (2)	
Smoking, n (%)				.20
Never	53 (62)	23 (53)	29 (70)	
Current	27 (31)	18 (42)	9 (21)	
Former	6 (7)	2 (5)	4 (9)	
Salt (gram per day), n (%)				.53
<6	41 (49)	20 (51)	21 (48)	
6‐10	30 (36)	12 (31)	18 (41)	
>10	12 (15)	7 (18)	5 (11)	
History of hypertension, n (%)	75 (86)	35 (81)	40 (91)	.33
Duration of hypertension (years), mean (SD)	7.6 (6.8)	7.9 (7.9)	7.3 (5.9)	.75
Use of antihypertensive medications, n (%)	67 (77)	33 (77)	34 (77)	>.99
Diuretics, n (%)	19 (22)	4 (9)	15 (34)	.01
CCB^c^, n (%)	51 (59)	25 (58)	26 (59)	>.99
BB^d^, n (%)	4 (5)	1 (2)	3 (7)	.63
ACEI^e^, n (%)	3 (3)	1 (2)	2 (5)	>.99
ARB^f^, n (%)	9 (10)	6 (14)	3 (7)	.46
Diabetes, n (%)	16 (18)	4 (9)	12 (27)	.06
Hyperlipemia, n (%)	16 (31)	4 (31)	12 (32)	>.99
Cerebral hemorrhage, n (%)	2 (2)	0 (0)	2 (5)	.49
Cerebral infarction, n (%)	6 (7)	4 (9)	2 (5)	.65
Myocardial Infarction, n (%)	1 (1)	1 (2)	0 (0)	.99
Angina, n (%)	3 (3)	0 (0)	3 (7)	.25
Heart failure, n (%)	4 (5)	2 (5)	2 (5)	>.99
Symptom^g^, n (%)	31 (36)	14 (33)	17 (39)	.71

a Not applicable.

b BP: blood pressure

c CCB: calcium channel blockers.

d BB: beta blockers.

e ACEI: angiotensin-converting enzyme inhibitors.

f ARB: angiotensin receptor blockers.

g Symptoms include dizziness, headache, tinnitus, fatigue, and edema of both lower limbs.

### Primary End Point

With a follow-up of 24 weeks, a higher percentage of participants (25/44, 57%) in the digital intervention group achieved controlled BP compared to the control group (21/43, 49%), although this difference was not statistically significant (*P*=.60; Table 2). Logistic regression analysis showed that the digital intervention did not significantly increase the hypertension control rate (odds ratio 0.73, 95% CI 0.31‐1.69; *P*=.46). In the digital intervention group, SBP decreased from 158.0 (SD 18.4) mm Hg at baseline to 137.5 (SD 13.0) mm Hg at 24 weeks, and DBP decreased from 93.8 (SD 10.3) mm Hg to 85.3 (SD 11.6) mm Hg. In the control group during that same period, SBP decreased from 161.1 (SD 18.2) mm Hg to 139.6 (SD 13.2) mm Hg, and DBP decreased from 99.2 (SD 9.2) mm Hg to 83.4 (SD 12.1) mm Hg.

**Table 2.** Hypertension control rate during follow-up in intervention and control groups.

Variable	Control group (n=43)	Digital intervention group (n=44)	Total (N=87)	*P* value^a^
Blood pressure control status, n (%)				.60
Controlled	21 (49)	25 (57)	46 (53)	
Uncontrolled	22 (51)	19 (43)	41 (47)	

a *χ*2_1_=0.282.

### Secondary End Point

**Figure 3.**
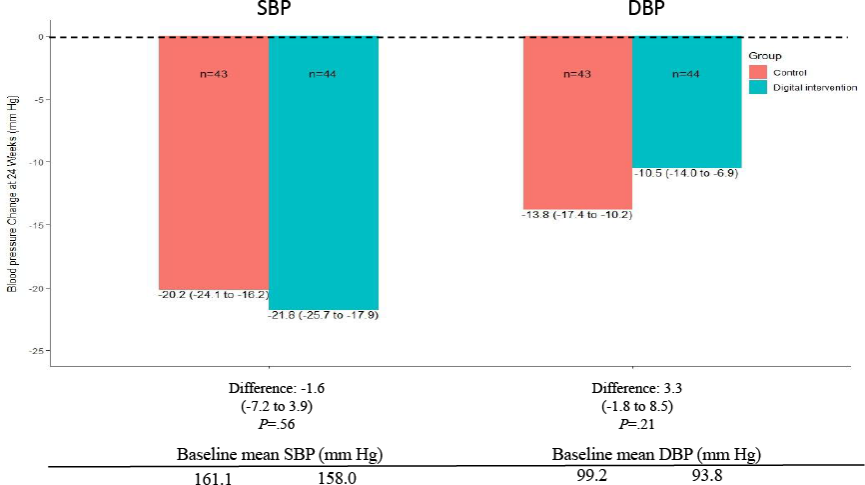
Changes from baseline to 24 weeks in SBP and DBP. Values are reported as mean (95% CI). DBP: diastolic blood pressure; SBP: systolic blood pressure.

The average reductions in SBP and DBP from baseline to 24 weeks were both significantly greater in the digital intervention group (SBP −21.8 mm Hg, 95% CI −25.7 to −17.9; and DBP −10.5 mm Hg, 95% CI −14.0 to −6.9) and the control group (SBP −20.2 mm Hg, 95% CI −24.1 to −16.2; and DBP −13.8 mm Hg, 95% CI −17.4 to −10.2). After adjusting for baseline SBP or DBP, the mean change from baseline to 24 weeks was comparable between the digital intervention and control groups for both SBP (between-group difference −1.6, 95% CI −7.2 to 3.9; *P*=.56) and DBP (between-group difference 3.3, 95% CI −1.8 to 8.5; *P*=.21; Figure 3).

Repeated measures analysis revealed that there was a substantial difference in SBP from baseline to 24 weeks in the 2 groups (between-time difference −21.0, 95% CI −25.3 to −16.7; *P*<.001), but without consideration of measurement time, no significant difference in SBP was observed between the digital intervention and control groups (between-group difference −2.6, 95% CI −7.8 to 2.6; *P*=.33). Due to the interaction between measurement time and group (*P*=.01), the amplitude of the change in DBP from baseline to 24 weeks differed between the 2 groups (the digital intervention group: between-time difference −8.5, 95% CI −12.6 to −4.4; *P*<.001; and the control group: between-time difference −15.9, 95% CI −20.0 to −11.7; *P*<.001). Additionally, baseline DBP was notably different between the 2 groups (between-group difference −5.4, 95% CI −9.6 to −1.3; *P*=.01), but no significant difference in DBP at 24 weeks was found (between-group difference 1.9, 95% CI −3.1 to 7.0; *P*=.45; Figure S1 in Multimedia Appendix 5).

### Adverse Events

Three adverse events were reported across the two groups: 1 participant in the digital intervention group was hospitalized for uncontrolled hypertension, whereas 2 participants in the control group were hospitalized—one for uncontrolled hypertension and the other for acute cervical trauma. Hypotension did not occur in either group. None of the adverse events were considered to be related to the digital intervention system.

## Discussion

### Principal Findings

The main finding of this study was that while the digital intervention did not show statistically significant results, it still played a role in improving BP control. Moreover, providing convenient BP measurements and health education to patients in rural areas substantially improved the hypertension control rate, achieving 57% in the digital intervention group versus 49% in the control group at 24 weeks. Notably, both the digital intervention and control groups exhibited considerable BP reduction. Specifically, the digital intervention group showed a decrease of 21.8 mmHg in SBP and 10.5 mmHg in DBP, whereas the control group experienced a reduction of 20.2 mmHg in SBP and 13.8 mmHg in DBP, respectively.

In China, the control rate of hypertension in rural areas is low, with previous studies reporting <10% [2]. The low hypertension control rate may partially be attributed to a lack of awareness among patients about high BP. Our study findings suggested that self-monitoring of BP and health education could motivate patients to take an active role in managing their hypertension. As demonstrated in meta-analyses, self-monitoring of BP, either alone or in conjunction with co-interventions, has been shown to reduce BP in patients with hypertension [17-19]. A prospective, randomized controlled study suggested that self-BP measurement as an adjunct to office-based BP monitoring improved treatment adherence [20]. While some studies have reported inconsistent results regarding BP reduction and adherence improvement [21,22], several trials still support the benefits of self-measured BP in improving hypertension awareness and control, and it is more effective in improving BP control when combined with education and counseling [23,24]. As home BP monitoring requires active patient participation, it may positively impact patients’ perception of their hypertension, thereby promoting adherence to lifestyle modifications and antihypertensive therapy. Notably, the use of home BP monitoring is recommended by hypertension management guidelines [13,25]. This study highlights the importance of providing free and accessible BP self-monitoring tools for all patients in need. Furthermore, a cluster-randomized trial in China compared nonphysician community health provider–led BP intervention with usual care, indicating that nonphysician community health providers received appropriate training and patients received affordable or free antihypertensive medications, remarkably improved the hypertension control in low-resource settings [26,27]. Therefore, a multifaceted strategy should be promoted in rural areas to reduce the morbidity and mortality associated with hypertension.

Digital therapeutics represents a promising approach to support the management of hypertension [28]. However, previous studies have demonstrated the inconsistent results of digital intervention in managing hypertension. In the study by Kario et al [9], 390 patients with essential hypertension who were not using antihypertensive medication were enrolled. The study found that the digital intervention group (HERB system: an interactive smartphone app [HERB Mobile] with a web-based patient management console [HERB Console] + standard lifestyle modifications) had a significantly greater reduction in 24-hour ambulatory, home, and office SBP at 12 weeks compared to the control group (standard lifestyle modifications only). Another randomized controlled trial from the United Kingdom included 622 hypertensive patients who had been treated but had poor BP control. The digital interventions consisted of self-monitoring of BP, patient education, promotion of healthy lifestyles, and medication guidance. After 12 months, the average BP in the digital intervention group decreased from 151.7/86.4 mm Hg to 138.4/80.2 mm Hg, whereas in the usual care group, it decreased from 151.6/85.3 mm Hg to 141.8/79.8 mm Hg, indicating that digital interventions led to better control of SBP than conventional care [29]. However, a randomized, open-label trial of 297 adults with uncontrolled hypertension showed that while participants randomized to a smartphone coaching app had greater self-confidence in controlling BP, they did not have lower BP at 6 months compared with those receiving a BP tracking app [30]. Conducted in real-world clinical practices in low-resource settings, our study demonstrated that the effectiveness of digital interventions was limited. This may be due to the relatively low educational levels and older age of the study participants, most of whom had only a primary school education, and the mean age of the digital intervention group was 62 years.

Although the role of digital interventions in BP management was not significant in this study, it still holds some implications for hypertension control in rural areas. First, self-measurement of BP and health education should be widely promoted in rural areas to increase patients’ self-awareness in managing their BP. Second, the effectiveness of digital interventions was limited among populations with lower educational levels and older age groups. It is important to better understand the barriers to using digital interventions faced by these individuals. Information on which patients would benefit most from a digital therapeutic intervention would be valuable for targeting the intervention to those patients. In addition, investment in targeted measures is necessary to enable the most vulnerable groups to participate in digital health initiatives and reduce the risk of digital health exacerbating health disparities.

### Limitations

Although we conducted a sample size calculation, the hypertension control rates in the 2 groups were quite similar. A larger sample size or an extended follow-up period may be needed to better understand the effects of the digital intervention. In addition, some patients measured their BP at home during follow-up, and we applied the same criteria as for office BP to determine whether their BP was controlled, whereas current guidelines recommend a difference between the home BP target and office BP target [13]. Moreover, while in-person clinic follow-up was the preferred method, some patients were unable to attend due to various barriers, particularly transportation difficulties. For these individuals, telephone follow-up was provided as an alternative. It is conceivable that these differing follow-up modalities could have impacted the results. Furthermore, measurement errors may have occurred because some patients self-monitored their BP rather than having it uniformly measured by a physician. Beyond these, the study design presented a potential for selection bias, as participation in the intervention group required the ability to use the WeChat mini-program.

### Conclusions

Our study demonstrated that digital interventions increased hypertension control rates in rural areas, although this improvement was not statistically significant. Nevertheless, providing convenient BP measurements and health education to these patients notably enhanced hypertension control rates. Further studies are necessary to validate the efficacy of these interventions.

## Supplementary material

10.2196/72416 Multimedia Appendix 1 Informed consent form.

10.2196/72416 Multimedia Appendix 2 Multiple screenshots of the mini-program.

10.2196/72416 Multimedia Appendix 3 The source code of mini-program.

10.2196/72416 Multimedia Appendix 4 Ethics review.

10.2196/72416 Multimedia Appendix 5 Selected baseline characteristics of the study participants. Changes in systolic blood pressure and diastolic blood pressure from baseline to 24 weeks.

10.2196/72416 Checklist 1 CONSORT-EHEALTH checklist (version 1.6.1).

## Abbreviations

BP: blood pressure
CCB: calcium channel blocker
CONSORT-EHEALTH: Consolidated Standards for Reporting Clinical Trials of Electronic and Mobile Health Applications and Online Telehealth
DBP: diastolic blood pressure
GAD-7: 7-item Generalized Anxiety Disorder
HBPM: home blood pressure monitoring
NYHA: New York Heart Association
OR: odds ratio
PHQ-9: 9-item Patient Health Questionnaire
SBP: systolic blood pressure
