# Supplementary material for: Effect of a Mobile Health Intervention in the Management of Hypertension: Open-Label Cluster-Randomized Trial
Source: JMIR Mhealth Uhealth. 2025 Dec 24;13:e72416. doi: 10.2196/72416 (PMC12735645; doi:10.2196/72416)
Supplement: Multimedia Appendix 1 [file mhealth-v13-e72416-s001.doc]

**Informed Consent Form**

**Dear Sir/Madam,**

We cordially invite you to participate in the research project titled "Establishing a Rural Hypertension Management Platform Based on a WeChat Mini Program". This study has been approved by the Sichuan Provincial People's Hospital Foundation (Project Source) and will be conducted collaboratively in eight natural villages. The study is led by Dr. Xiaoli Zhao, an attending physician in the Department of Cardiovascular Medicine.

This study has been reviewed and approved by the Medical Ethics Committee of the Sichuan Academy of Medical Sciences & Sichuan Provincial People's Hospital, with the ethics review approval number: Ethics Review (Research) No. 47, 2024.

**Why conduct this study?**

Hypertension has a high prevalence and low control rate, posing a serious threat to public health. Although existing medications and the promotion of healthy lifestyles can effectively control hypertension and significantly reduce mortality and disability rates, the awareness and control rates of hypertension remain low in our country. Clinical studies worldwide have demonstrated the effectiveness of digital interventions in hypertension management. However, there is currently no scientifically evaluated, widely applicable, and sustainable management tool that actively involves both healthcare providers and patients. To address this gap, we have developed a user-friendly WeChat Mini Program to assist rural doctors in managing blood pressure for hypertensive patients. This initiative aims to enhance the hypertension control rate in rural areas, improve patient prognosis, and provide a novel management approach for hypertension prevention and control.

**Who is (not) eligible to participate in the study?**

Inclusion criteria: 1) Age ≥18 years; 2) Individuals with diagnosed essential hypertension that remains uncontrolled; 3) Individuals with a mean baseline BP (calculated from the second and third readings): SBP ≥140 mmHg and/or DBP ≥90 mmHg; 4) For participation in the digital intervention, individuals should be willing to engage in self-monitoring and be able to use the WeChat mini-program, or have family members who could assist with its use.

Exclusion criteria: 1) Patients with NYHA Class IV heart failure; 2) Individuals who had experienced an acute cardiovascular event and stroke within the previous three month; 3) Patients with advanced-stage cancer; 4) Individuals planning for pregnancy, currently pregnant, or breastfeeding; 5) Individuals unable to use the WeChat mini-program even after training.

**What is required if you participate in the study?**

**Screening period:**

During the screening phase, you will be assessed based on inclusion and exclusion criteria. If you meet the eligibility criteria, you may voluntarily choose to participate in the study by signing an informed consent form.

We will collect your basic information, including:

Demographics: Age, gender, height, weight

Medical history: Hypertension, diabetes, coronary artery disease, heart failure, chronic kidney disease, stroke, etc.

Personal history: Smoking history, alcohol consumption, allergy history, dietary intake of vegetables and fruits, physical activity.

Medication usage: Antihypertensive drugs, antidiabetic drugs, statins, antiplatelet agents.

Mental health assessment: GAD-7 (Generalized Anxiety Disorder screening), PHQ-9 (Depression screening), Pittsburgh Sleep Quality Index.

This information will help determine the most appropriate hypertension treatment for you, and it will be accessible only to your doctor.

If you decide not to participate in the study, you are free to choose your preferred treatment approach.

**Intervention period:**

If you choose to participate in this study, you will receive a free blood pressure monitor for self-monitoring. Additionally, you will have a 50% chance of being assigned to the digital intervention group.

**Digital intervention group:**

If assigned to this group, you will receive the following interventions:

Use of the WeChat Mini Program "eKongya":

You will register as a patient and enter your basic information and assessment scales.

The mini-program will provide hypertension-related health education, online consultation, and promote self-blood pressure monitoring.

Rural doctors will offer online guidance, and you can track lifestyle changes within the program.

Blood pressure monitoring requirements:

You will measure your morning blood pressure at least twice on seven different days per month (with at least a 5-minute interval between two consecutive measurements) and entered into the WeChat Mini Program. If the average home blood pressure remained within target levels for three consecutive months, the frequency of measurements could be reduced to twice on three days each month.

Hypertension treatment: Your treatment will follow the latest hypertension guidelines, including lifestyle modifications and medication initiation if necessary.

**Control group:**

If assigned to this group, you will receive:

A printed hypertension education booklet and a free blood pressure monitor.

The option to monitor and record your blood pressure at your discretion.

The freedom to visit a hospital and take antihypertensive medication based on your personal preference.

**Follow-up period:**

At 6 months after the start of your treatment, we will conduct regular follow-ups, including:

Measurements of your blood pressure.

Recording of newly diagnosed conditions such as stroke, myocardial infarction, angina, and heart failure.

**What are the benefits of participating in the study?**

By participating in this study, you may experience improved blood pressure control and adopt a healthier lifestyle. Additionally, you will gain a better understanding of the prevention and treatment of hypertension and other cardiovascular diseases.

**What are the risks of participating in the study?**

There are no known adverse effects or risks associated with participating in this study.

If you experience any discomfort, changes in your condition, or any unexpected events during the study, regardless of whether they are related to the study, you should promptly inform your doctor. Your doctor will assess the situation and provide appropriate medical care.

Additionally, you will be required to regularly measure your blood pressure and enter the data into the WeChat Mini Program, which may take some of your time. However, this is crucial for effective blood pressure management.

**Do I need to pay any fees to participate in the study?**

The use of the WeChat Mini Program and the blood pressure monitor provided for blood pressure monitoring are completely free of charge. If you are assigned to the digital intervention group, you will also receive free access to the digital intervention within the WeChat Mini Program.

However, the cost of antihypertensive medications and any other medications needed for coexisting conditions will be the responsibility of the participant.

**Is my personal information confidential?**

Any information you provide in the WeChat Mini Program will be strictly confidential. Only the researchers, research oversight authorities, and the ethics committee are authorized to access your information records. Any public reports related to the results of this study will not disclose your personal identity. We will make every effort to protect the privacy of your personal medical information, within the limits allowed by law.

**What are the treatment options if I do not participate in the study?**

Participation in this study is entirely voluntary. You have the right to refuse to participate or to withdraw from the study at any time during the research process. This will not affect your relationship with your doctor or result in any loss of medical care or other benefits.

**Do I have to participate in the study?**

Participation in this study is completely voluntary. You may choose to decline participation or withdraw from the study at any time during the research process. This will not affect your treatment by your doctor. If you decide to withdraw, please contact your doctor, and you may be asked to undergo relevant examinations, which will be beneficial for protecting your health.

If you have any questions regarding your personal rights, you can contact the Ethics Committee of this hospital at 028-87393449.

**Participant declaration:**

I have read the above introduction to the study and fully understand the potential risks and benefits of participating in this research. I voluntarily agree to participate in this study.

I agree □ / disagree □ to allow other research studies to use my medical records and clinical specimens related to this study.

**Research participant's signature**： Date：＿ ＿ ＿ ＿

Research Participant's contact number: Telephone number:

(If applicable) Guardian/witness signature： Date：＿ ＿ ＿ ＿

Guardian/witness contact number： 　　 Telephone number:

**Researcher declaration:**

I confirm that I have explained the details of this study to the participant, particularly the potential risks and benefits of participation, and have answered all questions raised by the participant. The participant has voluntarily agreed to participate in this study. This informed consent form is made in two copies, with one signed copy retained by the researcher and one by the participant.

Research doctor's signature: 　　 Date：＿ ＿ ＿

Research doctor's work phone number: 　Telephone number:
