# Supplementary figures and images for: Effect of a Mobile Health Intervention in the Management of Hypertension: Open-Label Cluster-Randomized Trial
Source: JMIR Mhealth Uhealth. 2025 Dec 24;13:e72416. doi: 10.2196/72416 (PMC12735645; doi:10.2196/72416)

# Multiple screenshots of the mini-program

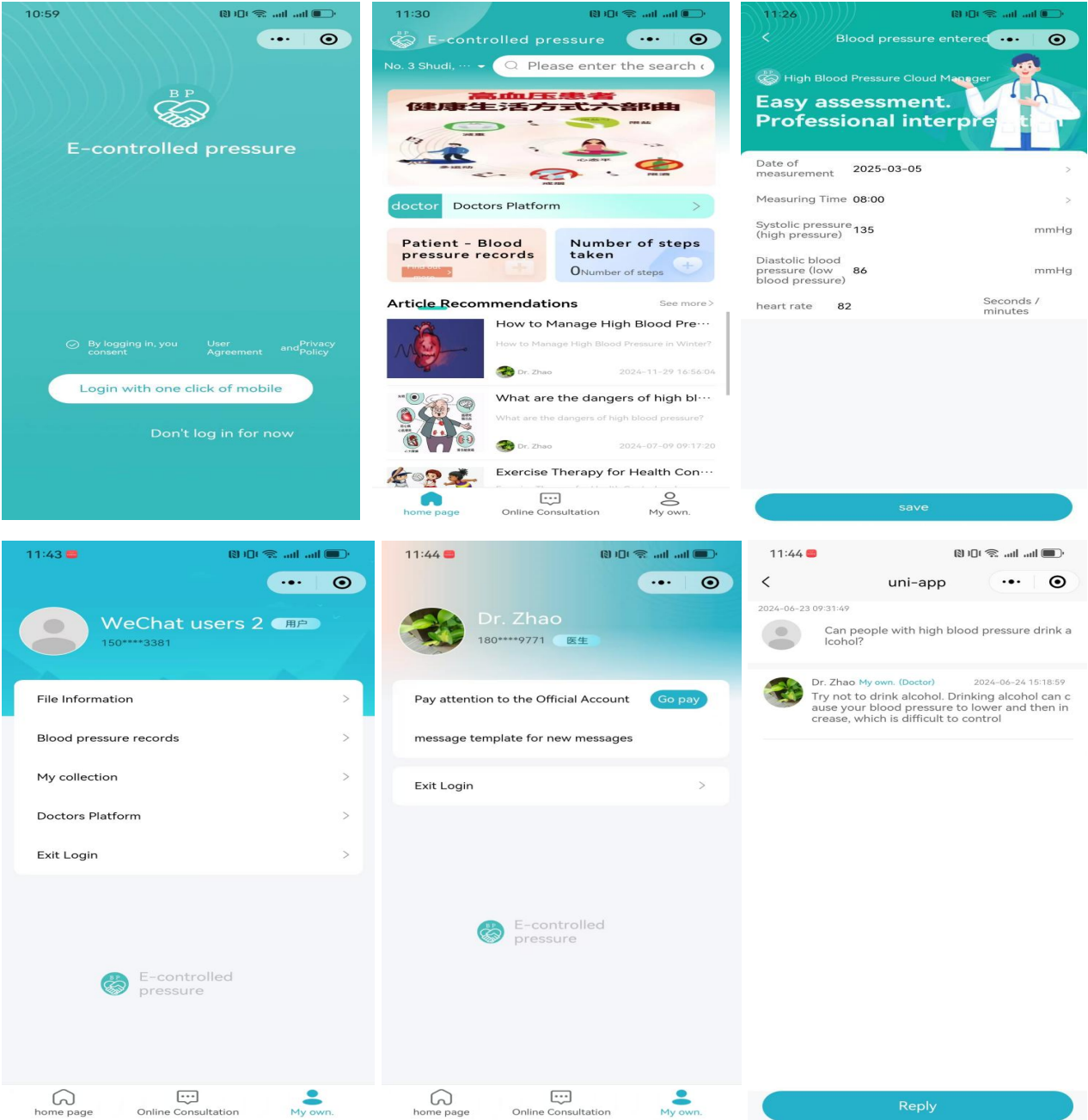

Supplement: Multimedia Appendix 2 [file mhealth-v13-e72416-s002.pdf]

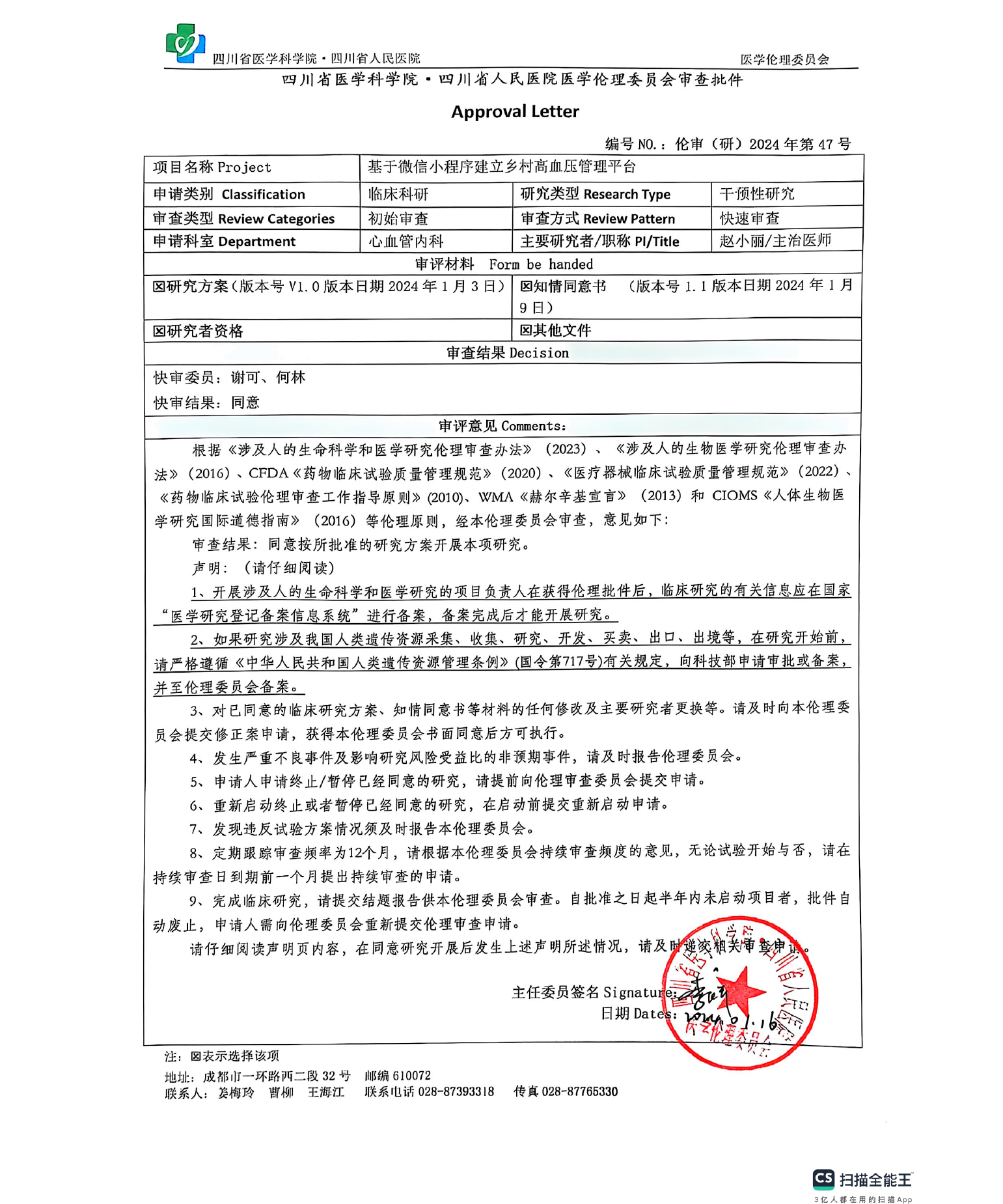

Supplement: Multimedia Appendix 4 [file mhealth-v13-e72416-s004.png]
