# Supplementary material for: Effect of a Mobile Health Intervention in the Management of Hypertension: Open-Label Cluster-Randomized Trial
Source: JMIR Mhealth Uhealth. 2025 Dec 24;13:e72416. doi: 10.2196/72416 (PMC12735645; doi:10.2196/72416)
Supplement: Multimedia Appendix 5 [file mhealth-v13-e72416-s005.docx]

Online-Only Supplemental Material

Table S1 Selected baseline characteristics of the study participants

Figure S1 Changes in systolic blood pressure and diastolic blood pressure from baseline to 24 weeks. Values are reported as mean and standard error (bars)

**Table S1 Selected baseline characteristics of study participants**

|  | Overall | **Control** | **Intervention** | P value |
| --- | --- | --- | --- | --- |
| Participants, n (%) | 87 (100) | 43 (100) | 44 (100) | —^a^ |
| Characteristics of participants | | | | |
| Hypertension family history, n (%) | 29 (33) | 13 (30) | 16 (36) | .71 |
| Moderate to high intensity physical activity per week, n (%) |  |  |  | .54 |
| ＜75 minutes | 28 (34) | 12 (30) | 16 (38) |  |
| 75—150 minutes | 12 (15) | 5 (13) | 7 (17) |  |
| ＞150 minutes | 42 (51) | 23 (58) | 19 (45) |  |
| Fruits (gram per day), n (%) |  |  |  | <.001 |
| <100 | 55 (66) | 34 (87) | 21 (48) |  |
| 100-250 | 26 (31) | 5 (13) | 21 (48) |  |
| >250 | 2 (2) | 0 (0) | 2 (5) |  |
| Vegetables (gram per day), n (%) |  |  |  | .004 |
| <100 | 21 (26) | 15 (41) | 6 (14) |  |
| 100-250 | 34 (42) | 9 (24) | 25 (57) |  |
| >250 | 26 (32) | 13 (35) | 13 (30) |  |
| Lipid-lowering drugs, n (%) | 9 (10) | 4 (9) | 5 (11) | 1.00 |
| Aspirin, n (%) | 5 (6) | 1 (2) | 4 (9) | .37 |
| GAD-7^b^, median (range) | 0.0 [0.0, 2.0] | 0.0 [0.0, 1.0] | 0.0 [0.0, 2.0] | .54 |
| PHQ-9^c^, median (range) | 0.0 [0.0, 4.0] | 0.0 [0.0, 0.5] | 3.0 [0.0, 8.0] | <.001 |
| PSQI^d^, median (range) | 1.0 [0.0, 7.0] | 7.0 [2.0, 14.0] | 0.0 [0.0, 0.0] | <.001 |

^a^Not applicable.

^b^GAD-7: Generalized Anxiety Disorder 7-item scale Anxiety Screening Scale.

^c^PHQ-9: Patient Health Questionnaire-9 item Depression Screening Scale.

^d^PSQI: Pittsburgh Sleep Quality Index.

**Figure S1. Changes in systolic blood pressure and diastolic blood pressure from baseline to 24 weeks. Values are reported as mean and standard error (bars).**

**
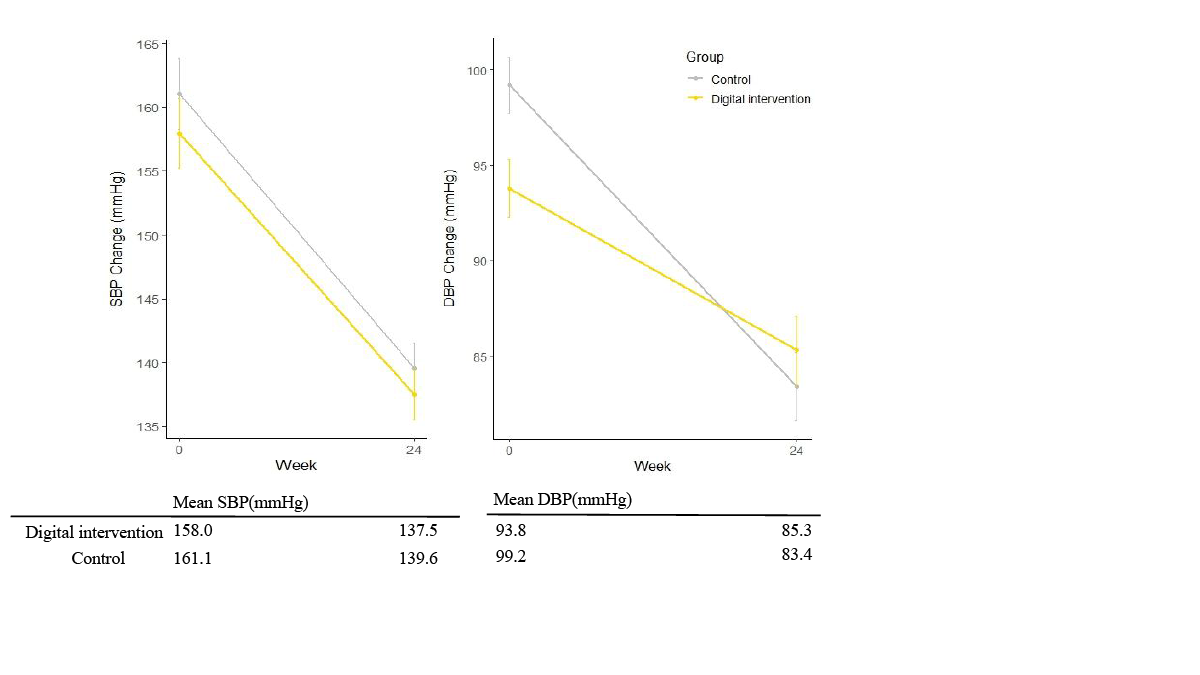
**
